# Genetic variation in the oxytocin receptor (*OXTR*) gene is associated with Asperger Syndrome

**DOI:** 10.1186/2040-2392-5-48

**Published:** 2014-09-16

**Authors:** Agnese Di Napoli, Varun Warrier, Simon Baron-Cohen, Bhismadev Chakrabarti

**Affiliations:** Autism Research Centre, Department of Psychiatry, University of Cambridge, Douglas House, 18B Trumpington Road, Cambridge, CB2 8AH UK; Cambridgeshire and Peterborough NHS Foundation Trust, CLASS Clinic, Cambridge, Elizabeth House, Fulbourn Hospital, Cambridge, CB21 5EF UK; Centre for Integrative Neurosciences and Neurodynamics, School of Psychology and Clinical Language Sciences, University of Reading, Reading, RG6 6AL UK

**Keywords:** Autism Spectrum Conditions (ASC), Asperger Syndrome (AS), Oxytocin receptor (OXTR), Haplotype analysis

## Abstract

**Background:**

Autism Spectrum Conditions (ASC) are a group of neurodevelopmental conditions characterized by impairments in communication and social interaction, alongside unusually repetitive behaviors and narrow interests. ASC are highly heritable and have complex patterns of inheritance where multiple genes are involved, alongside environmental and epigenetic factors. Asperger Syndrome (AS) is a subgroup of these conditions, where there is no history of language or cognitive delay. Animal models suggest a role for oxytocin (*OXT*) and oxytocin receptor (*OXTR*) genes in social-emotional behaviors, and several studies indicate that the oxytocin/oxytocin receptor system is altered in individuals with ASC. Previous studies have reported associations between genetic variations in the *OXTR* gene and ASC.

**Methods:**

The present study tested for an association between nine single nucleotide polymorphisms (SNPs) in the *OXTR* gene and AS in 530 individuals of Caucasian origin, using SNP association test and haplotype analysis.

**Results:**

There was a significant association between rs2268493 in *OXTR* and AS. Multiple haplotypes that include this SNP (rs2268493-rs2254298, rs2268490-rs2268493-rs2254298, rs2268493-rs2254298-rs53576, rs237885-rs2268490-rs2268493-rs2254298, rs2268490-rs2268493-rs2254298-rs53576) were also associated with AS. rs2268493 has been previously associated with ASC and putatively alters several transcription factor-binding sites and regulates chromatin states, either directly or through other variants in linkage disequilibrium (LD).

**Conclusions:**

This study reports a significant association of the sequence variant rs2268493 in the *OXTR* gene and associated haplotypes with AS.

## Background

Autism Spectrum Conditions (ASC) are a group of neurodevelopmental conditions characterized by difficulties in social interaction and communication, alongside unusually repetitive behaviors and narrow interests. Classic autism and Asperger Syndrome (AS) are two subgroups of ASC. They differ in that AS (unlike classic autism) has no history of language or cognitive delay [1]. The prevalence of ASC in the general population is around 1% [2], with a male:female sex ratio of 4:1 in classic autism [3], increasing to as high as 9:1 in AS [4]. ASC are highly heritable, as indicated in three different twin studies [5–7]. Despite the high heritability, they are characterized by complex patterns of inheritance where multiple genes, epigenetic and environmental factors are involved. Many loci have been implicated in the predisposition to these conditions [8–10], but only a few of these loci have been well-replicated.

The oxytocin receptor (*OXTR*) gene encodes a member of the class I family of G protein-coupled receptors, which contains seven transmembrane domains. It occupies 17 Kb on chromosome 3p25 and includes four exons and three introns. It is mainly expressed in the mammalian reproductive system and brain (pre-limbic circuits, nucleus accumbens, thalamus and amygdala) [11–13]. Oxytocin receptor (OXTR) regulates the activity of oxytocin (OXT), a neuropeptide implicated in labor, uterine contraction and lactation. OXT is also involved in the establishment of maternal [14] and social [15] behaviors. The *OXT* and *OXTR* genes are good candidate genes for studying the genetic basis of ASC, because of their role in social-emotional behaviors [16–19]. Intranasal inhalation of OXT represents a potential treatment of ASC, improving social interaction [20] and emotion recognition [21].

A previous genetic study from our group identified a nominal association between the single nucleotide polymorphism (SNP) rs237880 in *OXTR* and autistic traits [22]. Several other studies reported an association between genetic variations in *OXTR* and ASC or related phenotypes. An association study in a Chinese population found that two SNPs (rs2254298 and rs53576) and haplotypes, including rs53576, are involved in the predisposition to ASC [23]. These findings were partially replicated in Japanese [24] and Caucasian [25] populations. Yrigollen *et al*. [26] identified an association between rs2268493 and affiliative behavior in ASC in a largely (93%) Caucasian sample, while Campbell *et al*. [27] reported three SNPs (rs2268493, rs1042778, rs7632287) were nominally associated with ASC and related phenotypes. Another study conducted in a mixed-ethnicity population (84% Caucasian, 4.9% African-Canadian, 11.1% ‘other’) found that a genetic variation (rs237885) and one haplotype (rs237885-2368493) in *OXTR* were associated with callous-unemotional traits, which are related to reduced empathy [28]. Two other studies reported an association between genetic variations in *OXTR* and autism-related phenotypes: empathy [29] and mind-reading [30]. Finally epigenetic studies show that different methylation patterns in *OXTR* are involved in the predisposition to classic autism [31] and variability of social perception [32].

In the current study we tested for a possible association of nine SNPs in *OXTR* with AS, performing a case–control study in a Caucasian population sample. We hypothesized that the SNPs in *OXTR*, alone or in combination, would be associated with AS. We did not have a specific hypothesis about any particular SNP, and hence mapped the gene using multiple SNPs. This approach is useful in testing the role of a candidate gene (rather than a specific SNP), since it allows a test of genetic association directly and by proxy [33].

## Methods

### Ethical approval

The current study was approved by both the Cambridge Psychology Research Ethics Committee and the NHS Research Ethics Committee (United Kingdom). The research was consistent with the Declaration of Helsinki. All participants gave informed consent to take part in the study.

### Participants

All participants reported that they were of Caucasian origin for at least three generations (four Caucasian grandparents) and lived in northern Europe. They were recruited through the online database of Autism Research Centre in the University of Cambridge.

They were asked to complete the Autism Spectrum Quotient (AQ) online. The AQ is a measure of autistic traits and was used as a screen for cases and controls. Autistic traits are distributed continuously across the general population, where individuals with ASC represent one end of this continuum [34]. The AQ has high heritability in the general population [35] and individuals with AS have a mean AQ of 35.8 ± 6.5, while the mean AQ in the general population is 16.4 ± 6.3. An AQ score of 32+ is a useful cut-off for ASC but is not diagnostic [36]. A total of 530 participants were selected: 118 cases (74 males and 44 females) and 412 controls (185 males and 227 females). This sample of participants does not overlap with the sample reported in the previous genetic study of AS carried out in our laboratory [22]. The mean age for cases was 40.5 ± 15.3 years, and for controls was 28.7 ± 4.6 years. Age difference between the two groups is not a confounding factor in this study, as age is not correlated with AQ or a diagnosis of AS. All cases had been previously diagnosed with AS by clinicians (psychiatrists or clinical psychologists) in recognized clinics in the United Kingdom using the criteria of the Diagnostic and Statistical Manual of Mental Disorders, Fourth Edition, Text Revision (DSM-IV-TR) [1] or the International Statistical Classification of Diseases and Related Health Problems, Tenth Revision (ICD-10) [37]. Controls had no diagnosis of ASC or any other psychiatric condition and did not have first-degree relatives with an ASC diagnosis. The AQ was used to validate the AS diagnosis in cases (AQ score >32) and select controls (AQ score ≤24) from the low end of the distribution of autistic traits to avoid any risk of confounding controls with individuals who might have high autistic traits or undiagnosed ASC. Cases had a mean AQ of 35.6 ± 8.9 (range: 7 to 50, males mean: 35.1 ± 8.7, females mean: 36.6 ± 8.8) and controls had a mean AQ of 14.9 ± 5.0 (range: 2 to 23, males mean: 16.0 ± 4.4, females mean: 13.9 ± 5.1).

### Single nucleotide polymorphism selection

We selected nine SNPs in *OXTR* with a minor allele frequency (MAF) >0.05, as indicated by the dbSNP database and the HapMap genome browser, release 27 (operated by the National Institutes of Health [NIH], Bethesda, Maryland, United States) in the CEU (Utah residents with northern and western European ancestry) population. We chose SNPs located on chromosome 3 from 8795543 bp to 8810896 bp, to provide maximal coverage of *OXTR*. Inter-SNP distance was less than 5 Kb (GRCH37.p10 Primary Assembly, National Center for Biotechnology Information [NCBI]). The SNPs selected were available on the TaqMan SNP Genotyping Assays (Applied Biosystems Inc., California, United States) and rs237900 was a tag SNP (HapMap genome browser, release 27). rs2301261 (chr3: 8810896) and rs237885 (chr3: 8795543) are the most upstream and downstream SNPs on the gene, respectively (Table 1). Buccal swab kits were sent and returned by post and DNA was anonymized and extracted following a previously reported protocol [38]. SNP genotyping was performed using the TaqMan SNP genotyping assays as previously described [22]. None of the selected SNPs deviated from Hardy-Weinberg equilibrium, as tested using Plink v1.07 [39] at α = 0.05.

**Table 1:** **SNPs into the** ***OXTR*** **gene analyzed in the current study**

SNP ID	Chromosomal position (bp)	Derived/Ancestral Allele	MAF
rs237885	8795543	T/G	T = 0.438
rs2268490	8797085	T/C	T = 0.119
rs2268493	8800840	C/T	C = 0.227
rs2254298	8802228	A/G	A = 0.070
rs53576	8804371	A/G	A = 0.413
rs237894	8806531	C/G	C = 0.331
rs2268496	8808141	T/A	A = 0.222
rs237900	8808696	A/G	A = 0.406
rs2301261	8810896	A/G	A = 0.053

SNPs analyzed in this study are reported alongside their chromosomal position and MAF. Bp, base pair; MAF, minor allele frequency; SNP, single nucleotide polymorphism.

### Statistical analysis

#### Single nucleotide polymorphism association

SNP association test was performed using Plink v1.07, to test for possible association between nine SNPs in *OXTR* and AS. We conducted the test under the null hypothesis of no association between each SNP and AS, using a significant level of *P* <0.05. Bonferroni correction was performed after controlling for linkage disequilibrium (LD) between the selected SNPs through the SNPSpD web interface [40]. The estimated average number of independent loci was seven and α was estimated to be equal to 0.0073. Only *P* values below or equal to the SNPSpD α threshold were considered significant for single SNP association analysis.

#### Haplotype analysis

Haplotype analysis in the case–control study was performed using Plink v1.07. We conducted omnibus two-, three- and four-loci haplotype analyses using logistic regression, under the null hypothesis of no association between haplotypes and AS. Permutation correction (50,000 permutations) was used to correct the *P* values for family wise error rates (FWER). Permutation correction changes phenotype-genotype correlation but does not alter LD patterns between SNPs. Haplotypes with *P* values equal or below the α threshold of 0.05 were considered significant. LD blocks in our sample and in the CEU population were analyzed using Haploview 4.2 [41]. Haplotype analysis in the CEU population was performed using data reported in the HapMap genome browser, release 27.

#### Single nucleotide polymorphism annotation

Functional annotation of significantly associated SNPs was performed using multiple tools. HaploReg [42] was used to predict the presence of regulatory motifs and conservation sites and to evaluate chromatin states. F-SNP [43] provided an overall functional score for the genetic variations investigated. Previous genetic associations between the loci analyzed and ASC were detected through the Genetic Association Database [44]. Manual verification of the loci for functional roles was conducted using the University of California Santa Cruz (UCSC) genome browser [45]. Finally, SNPnexus provided information about structural variants and conservation sites in the loci analyzed [46].

## Results

### Single nucleotide polymorphism association

**Figure 1:**
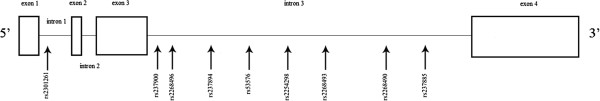
**Schematic representation of the** ***OXTR*** **gene.** The nine single nucleotide polymorphisms analyzed in the current study are shown by arrows.

SNP rs2268493 was associated with AS (Table 2). This genetic variation localizes in intron 3 of *OXTR* (see Figure 1) and showed a statistically significant association after Bonferroni correction for effective total number of SNPs (Table 2). None of the other SNPs were nominally associated with AS.

**Table 2:** **Results of SNP association between nine genotyped SNPs in the** ***OXTR*** **gene and AS**

SNP ID	Frequency Minor Allele (cases)	Frequency Minor Allele (controls)	OR	X ^2^	***P***value
rs237885	0.4612	0.458	1.013	0.0074	0.9317
rs2268490	0.1282	0.1382	0.9171	0.1535	0.6952
rs2268493	0.2155	0.3074	0.619	7.442	***0.00637***
rs2254298	0.0862	0.1284	0.6404	3.062	0.0802
rs53576	0.3559	0.3037	1.267	2.308	0.1287
rs237894	0.3248	0.267	1.321	2.992	0.0837
rs2268496	0.2179	0.2437	0.8648	0.6625	0.4157
rs237900	0.3846	0.3897	0.9787	0.0199	0.8879
rs2301261	0.0769	0.1078	0.6899	1.9	0.1681

Significant *P* values after Bonferroni correction for total number of SNPs are written in bold and italicized. AS, Asperger Syndrome; OR, odds ratio; SNP, single nucleotide polymorphism.

### Haplotype analysis

One two-loci haplotype (rs2268493-rs2254298), two three-loci haplotypes (rs2268490-rs2268493-rs2254298, rs2268493-rs2254298-rs53576) and two four-loci haplotypes (rs237885-rs2268490-rs2268493-rs2254298, rs2268490-rs2268493-rs2254298-rs53576) were associated with AS after permutation correction. All these haplotype combinations include rs2268493, which was associated with AS in the SNP association test (Table 3).

**Table 3:** **Results of haplotype analysis in the AS case**–**control study**

Number of SNPs	Haplotype combination	Chromosomal position	***P***value	FWER corrected ***P***value
2	rs2268493-rs2254298	8800840-8802228	0.00086	0.00468
3	rs2268490-rs2268493-rs2254298	8797085-8802228	0.000365	0.0018
3	rs2268493-rs2254298-rs53576	8800840-8804371	0.00208	0.01268
4	rs237885-rs2268490-rs2268493-rs2254298	8795543-8802228	0.0012	0.00712
4	rs2268490- rs2268493-rs2254298-rs53576	8797085-8804371	0.00132	0.0079

Haplotypes with significant *P* values after permutation correction are reported. AS, Asperger Syndrome; FWER, family-wise error rates; SNPs, single nucleotide polymorphisms.

**Figure 2:**
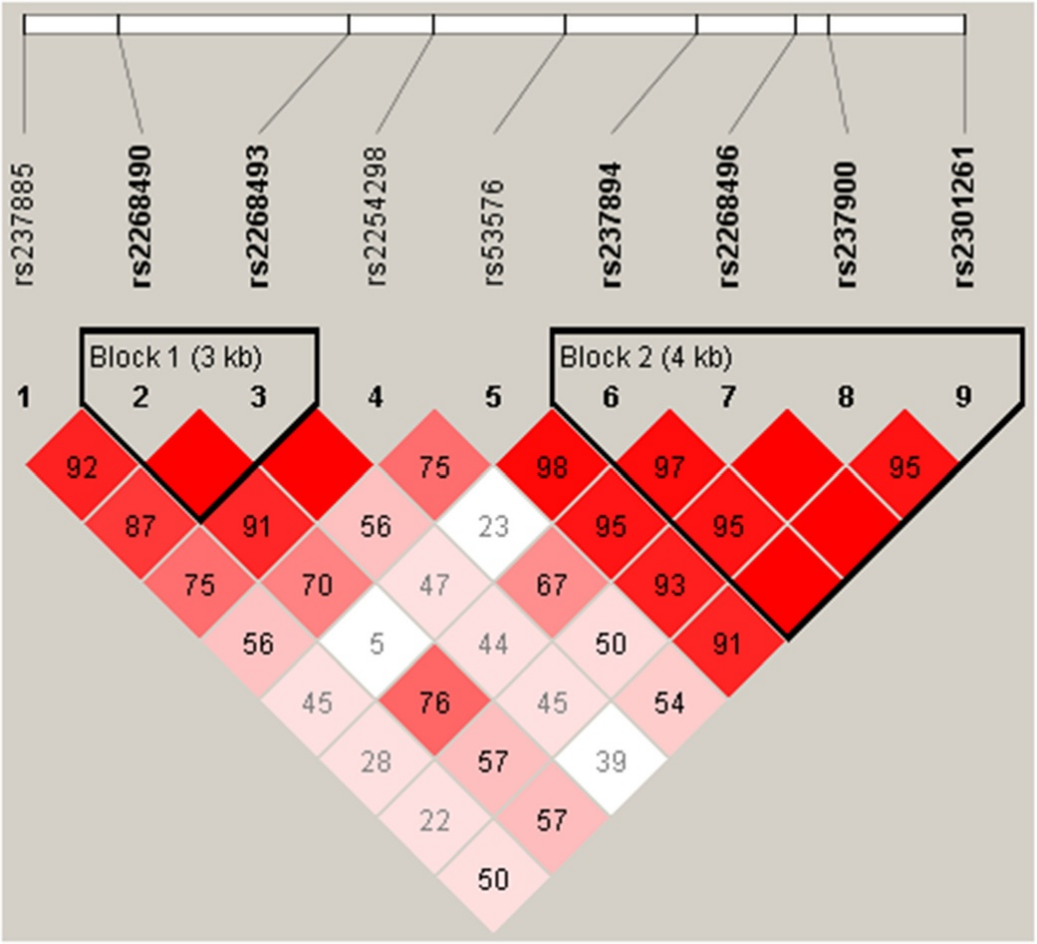
**Linkage disequilibrium plot of the** ***OXTR*** **gene calculated in the sample studied.** Numbers in the squares indicate D’ values.

**Figure 3:**
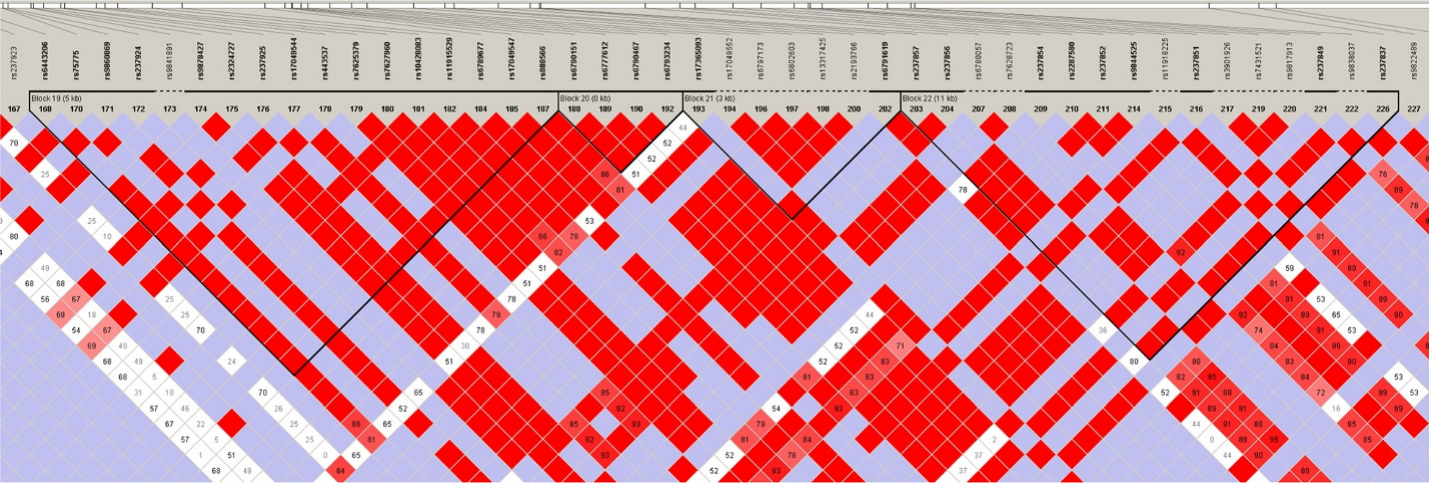
**Linkage disequilibrium plot of the** ***OXTR*** **gene calculated in the CEU (Utah residents with northern and western European ancestry) population (detail).** Numbers in the squares indicate D’ values.

In our sample rs2268493 and rs2268490 form a 3 Kb LD block (Block 1; Figure 2). These two variations are part of two 4-loci haplotype combinations associated with AS (rs2268490-rs2268493-rs2254298-rs53576 and rs237885-rs2268490-rs2268493-rs2254298) (Table 3). rs2268493 is included in an LD block in the CEU population (Block 20), alongside rs6790151, rs6777612, rs6790467 and rs6793234 (Figure 3).

## Discussion

In the present study we conducted a case–control study in a Caucasian sample to test for genetic association between nine SNPs in the *OXTR* gene and AS. Our results indicate a significant association between the common genetic variant rs2268493 in *OXTR* and AS. These corroborate previously reported association between SNPs in *OXTR* and individuals with an ASC more broadly. The two most reported SNPs in *OXTR* that are associated with ASC are rs2254298 and rs53576 [23–25], while rs2268493 has been significantly [26] and nominally [27] associated with ASC in two different studies.

In the current study we find a genetic association of rs2268493 with AS, which remains significant after Bonferroni correction. Haplotype analysis reports significant association of five haplotypes in *OXTR* with AS (rs2268493-rs2254298, rs2268490-rs2268493-rs2254298, rs2268493-rs2254298-rs53576, rs237885-rs2268490-rs2268493-rs2254298, rs2268490-rs2268493-rs2254298-rs53576). rs2268493 is included in all of these haplotype combinations and it localizes, along with rs2268490, in an LD block associated with AS in our sample. In the CEU population, rs2268493 localizes in an LD block with other four SNPs (rs6790151, rs6777612, rs6790467 and rs6793234).

rs2268493 is included in intron 3 of *OXTR* (Figure 1) and it localizes 5930 bp upstream of the intron 3-exon 4 splice junction. Functional annotation indicates that this genetic variation alters six different transcription factor-binding sites (F-SNP, HaploReg). Among them are SOX_3 and Pou3f3, which are involved in neural development [47, 48]. Moreover, rs2268493 and haplotypes that include this variation have been reported to be associated with ASC in earlier reports [26, 27]. *OXTR* has four splice variants and three of them are protein-coding; one transcript includes four exons and three introns, another variant is composed of the first three exons (1, 2 and 3) and introns 1 and 2, and the other two variants include exon 1, intron 1 and part of exon 2 (UCSC genome browser). Genetic variations that disrupt regulatory regions can affect gene expression in a time- and tissue-specific manner. Molecular studies are needed to understand this further. rs2268493 localizes in an LD block when calculated in the general population, where four other SNPs are included. Three SNPs (rs6777612, rs6790467 and rs6793234) alter regions that regulate the chromatin states in several neuronal cell lines (such as fetal brain and substantia nigra) (HaploReg).

This is the first study which reports a statistically significant association between one SNP (rs2268493) in *OXTR* with AS in a Caucasian sample, as other studies have focused on the whole spectrum of ASC. A previous study from our laboratory analyzed the genetic association between candidate genes (including *OXTR*) and AS [22] in an independent sample, while the current study represents a more in-depth analysis of the involvement of *OXTR* in AS. Campbell *et al.* have previously reported a nominally significant association between this SNP and a narrow ASC diagnosis in family-based samples [27]. However, another study by Li *et al.* failed to identify any association between the same SNP and ASC in a Japanese population [24]. Such differences can arise due to differences between populations studied, as well as differences in criteria for diagnoses between AS and other subgroups of ASC. It is currently unclear if these differences can be explained by a different genetic architecture underlying these conditions. One way to test this hypothesis is by investigating common variants in candidate genes in AS specifically.

A limitation of this study is the moderate sample size and so further studies are necessary to confirm our findings. Multiple genes are involved in the predisposition for ASC, interacting with epigenetic and environmental factors. For this reason a single polymorphism or haplotype is unlikely to be the sole cause of these conditions. Testing this SNP for association with endophenotypes related to ASC will be an interesting next step.

## Conclusions

In summary, we report a significant association between a common genetic variant in the *OXTR* gene (rs2268493) with AS. Five haplotypes that include this variant are also associated with AS. This provides further evidence suggesting a role of the oxytocinergic system in the aetiology of ASC, and shows that rs2268493 in *OXTR* is associated with AS, a high-functioning form of ASC.

## Authors’ information

ADN and VW are joint first authors; SBC and BC are joint senior authors.

## Abbreviations

AQ: Autism Spectrum Quotient
AS: Asperger Syndrome
ASC: Autism Spectrum Conditions
FWER: Family wise error rates
LD: Linkage disequilibrium
MAF: Minor allele frequency
OXT: Oxytocin
OXTR: Oxytocin receptor
SNP: Single nucleotide polymorphism.
